# A mouse model of ankle-subtalar joint complex instability induced post-traumatic osteoarthritis

**DOI:** 10.1186/s13018-021-02683-0

**Published:** 2021-09-01

**Authors:** Peixin Liu, Kaiwen Chen, Shuo Wang, Chunzhuo Hua, Hongtao Zhang, Jia Yu

**Affiliations:** 1 Department of Orthopedics, The First Affiliated Hospital of Soochow University, 899 Pinghai Road, Suzhou, 215006 Jiangsu People’s Republic of China; 2 Orthopedic Institute, Soochow University, 178 Ganjiangdong Rd, Suzhou, 215006 Jiangsu People’s Republic of China

**Keywords:** Ankle instability, Subtalar instability, Cervical ligament, Gait, Biomechanics

## Abstract

**Background:**

Ankle-subtalar joint complex instability is not uncommonly presented in the clinic, but symptoms and signs similar to other conditions can easily lead to its misdiagnosis. Due to the lack of appropriate animal models, research on ankle-subtalar joint complex instability is limited. The aims of the present study were to establish an animal model of ankle-subtalar joint complex instability in mice and to explore its relationship with post-traumatic osteoarthritis (PTOA).

**Methods:**

Twenty-one male C57BL/6J mice were randomly divided into three groups: SHAM group (sham surgery group), transected cervical ligament + anterior talofibular ligament (CL+ATFL) group, and transected cervical ligament + deltoid ligament (CL+DL) group. Two weeks after surgery, all mice underwent cage running training. Balance beam and gait tests were used to evaluate the changes in self-movement in the mice after ankle-subtalar ligament injury. Micro-CT and histological staining were used to evaluate the progress of PTOA.

**Results:**

Compared with the SHAM group, balance and gait were affected in the ligament transection group. Twelve weeks after surgery, the time required to cross the balance beam in the CL+ATFL group was 35.1% longer and the mice slipped 3.6-fold more often than before surgery, and the mean step length on the right side was 7.2% smaller than that in the SHAM group. The time required to cross the balance beam in the CL+DL group was 32.1% longer and the mice slipped 3-fold more often than prior to surgery, and the average step length on the right side was 5.6% smaller than that in the SHAM group. CT images indicated that 28.6% of the mice in the CL+DL group displayed dislocation of the talus. Tissue staining suggested that articular cartilage degeneration occurred in mice with ligament transection 12 weeks after surgery.

**Conclusions:**

Transected mice in the CL+ATFL and CL+DL groups displayed mechanical instability of the ankle-subtalar joint complex, and some mice in the CL+DL group also suffered from talus dislocation due to ligament injury leading to loss of stability of the bone structure. In addition, as time progressed, the articular cartilage displayed degenerative changes, which affected the ability of animals to move normally.

## Introduction

Acute ankle sprain is among the most common musculoskeletal injuries due to both sporting and general activities. Approximately 2 million acute ankle sprains occur each year in the USA with related healthcare costs as high as $4.2 billion annually [1–3]. Although approximately 25% of cases are joint injuries of the lateral ankle and subtalar joint or ankle-subtalar joint complex injuries, similar mechanisms of injury and clinical manifestations in the subtalar joint are often neglected, leading to undiagnosed or misdiagnosed ankle-subtalar joint complex injury [4]. Additionally, due to the lack of treatment, the probability of recurrent ankle sprain is increased resulting in chronic joint instability [5]. However, because of similar manifestations, it is difficult to diagnose whether ankle joint instability involves subtalar joint instability, so the incidence rate of ankle-subtalar joint complex instability is probably higher [6–8]. As instability occurs, the probability of joint degeneration increases, even affecting adjacent joints. A number of researchers have highlighted that severe ankle instability causes degeneration in the subtalar and talonavicular joints [9]. Even ankle arthrodesis may induce or exacerbate the progression of adjacent arthritis, especially in the subtalar joint [10]. Therefore, the subtalar joint is important when conducting ankle joint research.

The ankle-subtalar joint complex is the core of the hindfoot and plays a key role in the control of balance in both daily and sporting activities [11]. However, there is no consensus on the diagnosis and treatment of ankle-subtalar joint complex instability in the international community [12]. Although a number of researchers have demonstrated that subtalar joint instability is often accompanied by ankle instability, the structure of the ankle-subtalar joint complex is invariably studied in isolation to its function, with little attention paid to the subtalar joint [2, 13]. Therefore, no suitable animal model of the ankle-subtalar joint complex has so far been established. An animal model of ankle sprain was established for the first time in 2008. The Hubbard-Turner team subsequently demonstrated that mechanical instability could be induced in the ankle joint in this mouse model by removal of different ligaments on its lateral side, while Chang et al. demonstrated that the ankle joint in humans and mice are comparable through similar methods of modeling, providing a reliable animal model of osteoarthritis (OA) [14–16]. Such studies substantially promote the development of ankle joint research and also offer new concepts in translational orthopedics research of instability in the ankle-subtalar joint complex [17, 18].

Inspired by the mouse model of ankle instability, we hypothesized that transection of the appropriate ligaments could induce mechanical instability in the ankle-subtalar joint complex resulting in its degeneration. To test this hypothesis, we designed two mouse models that induce ankle-subtalar joint complex instability. The results of sensory-motor function (balance and gait) in the present study should determine the feasibility of the model and whether micro-CT and histological staining can demonstrate the progression of arthritis, providing a reference for clinical ligament repair.

## Methods

### Animals

Twenty-one male C57BL/6J mice (6 weeks old, 18 ± 2 g) were purchased from JOINN Laboratories (Suzhou), Inc. (License No. SCXK(Su) 2018-0006, Suzhou, China). The mice were initially maintained for 2 weeks prior to experimentation to allow them to adapt to the environment, then 5 days to train on the balance beam and the gait exercise. The experimental animals (3–4 mice/cage) were placed in an environment with a light/dark cycle of 12 h, and temperature and humidity of 22 ± 2 °C and 55 ± 10%, respectively. The mice had free access to food and water. The health of each mouse was checked every day.

### Surgical procedures

**Fig. 1:**
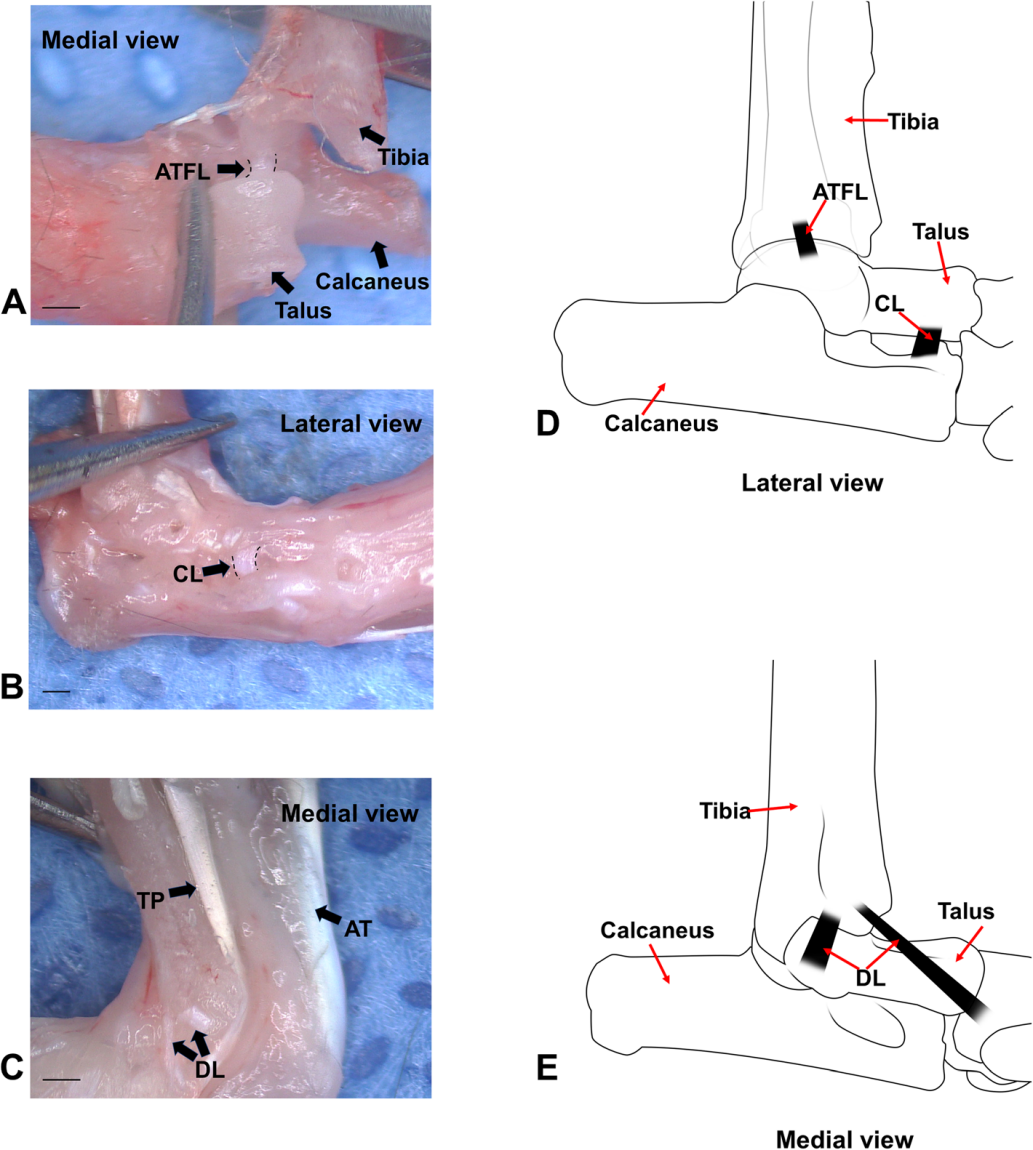
Anatomical structure and location of ankle ligament in mice. **A** Location of the anterior talofibular ligament (ATFL, ligament within the dotted lines), medial view. **B** Location of the cervical ligament (CL, ligament within the dotted lines), lateral view. **C** Medial structure of the foot and ankle. DL, deltoid ligament; TP, posterior tibial tendon, AT, Achilles tendon. **D** Schematic diagram of the anterior talofibular ligament (ATFL) and cervical ligament (CL). **E** Schematic diagram of the deltoid ligament (DL). Scale bar = 1 mm

At the age of 8 weeks, the mice were randomly divided into three groups. All mice were anesthetized by intraperitoneal injection of 4% chloral hydrate at a dose of 0.01 ml/g. The entire right foot and right leg were disinfected three times with balls of cotton wool soaked in 75% alcohol. After disinfection, the anesthetized mice were transferred to a microsurgical theater with a sterile surgical pad. For the transected cervical ligament + anterior talofibular ligament (CL+ATFL) group, an oblique downward longitudinal incision was created on the skin over the right ankle to expose the ankle joint. In front of the ankle joint, the ATFL connecting the body of the lateral talus and the lower edge of the fibula is then visible (Fig. 1A) which was transected with a scalpel. The fibular long and short tendons and the extensor digitorum longus tendons were separated with micro tweezers, exposing the anterior and middle articular surface of the subtalar joint, after which the CL was transected (Fig. 1B). In the transected cervical ligament + deltoid ligament (CL+DL) group, a vertical longitudinal incision was created on the medial skin of the ankle joint. The DL (Fig. 1C) was obtusely separated and transected from the anterior part of the medial malleolus to the scaphoid and talus. The CL was transected in the same manner as described above for the anterior ligament. The SHAM group underwent sham surgery at the corresponding position but without any ligaments being transected. The incision was washed with sterile saline then closed and disinfected with an Iodophor ball of cotton wool. No antibiotics or painkillers were used after surgery. The incision was disinfected with cotton wool balls soaked with Iodophor twice a day for a week.

### Balance measurement

Balance was evaluated by measuring the ability of the mice to reach a safe platform via an inclined (15°) beam. The crossbeam was 1 m long and 20 mm in diameter with one end connected to a camera clip and the other end connected to the opening of a cassette on the platform through which the mice could escape [15, 19–21]. The mice were trained to run from the end of the beam with the camera clip to the end with the cassette. A test was considered effective when each mouse had crossed the beam twice in a row, without pausing on the beam. During experimentation, each mouse completed two tests at the same time point. The duration required for the mouse to cross the beam and the number of times the right hind foot slipped off the beam were recorded as dependent variables. The balance test was performed prior to surgery, then 3 days and 1, 4, 8, and 12 weeks afterward. The mean value of the two durations at each time point was recorded.

### Footprint test

Prior to surgery and 12 weeks afterward, the front and hind feet of each mouse were coated with red and blue nontoxic pigments, respectively. The mice were placed on a U-shaped track, length 50 cm, width 10 cm, and height 10 cm, from which their footprints were obtained [15, 19]. The length of the right print of three consecutive hindfoot footprints was measured for each mouse, excluding the footprints left by the start and end gait. The mean value of each test was used for statistical analysis.

### Micro-CT

The mice were sacrificed 12 weeks after surgery, and the right ankle was harvested then fixed in 10% neutral formalin for 48 h. After fixation, the ankle was scanned by micro-CT (SkyScan 1176, Aartselaar, Belgium), with the following scanning parameters: 50 kV, 500 mA, at a resolution of 9 μm [22]. The scanned images were processed and recombined using the NRecon and DataView software. The cartilaginous layers of the ankle and subtalar joints and 10 consecutive layers from the region of interest (ROI) were selected for quantitative analysis in the CTAN software, then three-dimensional reconstruction was performed using the Mimics 15.0 software (Materialise, Belgium) [23].

### Histomorphometric analysis

After micro-CT imaging, all specimens were decalcified in 10% EDTA (pH = 7.4) for 1 month, after which the specimens were dehydrated through a gradient of increasing alcohol concentrations. The ankle specimens were then placed into n-butanol for 10 h and then immersed in paraffin for 7 h. The paraffin blocks were then sliced into 6-μm-thick coronal sections using a semi-automatic paraffin sectioning machine. The sections were stained with hematoxylin and eosin (HE) to evaluate the changes in the thickness of the cartilage in the ankle-subtalar joint complex, pathological changes were evaluated using a modified Mankin score [23, 24]. Changes in cartilage proteoglycan levels were determined using Safranin O-Fast Green staining, from which damage was evaluated using the OARSI OA cartilage histopathology evaluation system [23, 25, 26].

### Statistical analysis

SPSS v23.0 (SPSS Inc., Chicago, IL, USA) and GraphPad Prism 8.0 (GraphPad Software, La Jolla, CA, USA) were used for statistical analysis. Firstly, the normality of the distribution and the homogeneity of variance of each group of data were tested. Normally distributed data that had equal variance were analyzed by one-way ANOVA; otherwise, the data were analyzed using the Kruskal-Wallis test. The data are expressed as means ± standard deviation (SD). *P*-values < 0.05 of the differences between the two groups were considered statistically significant.

## Results

### Balance

**Fig. 2:**
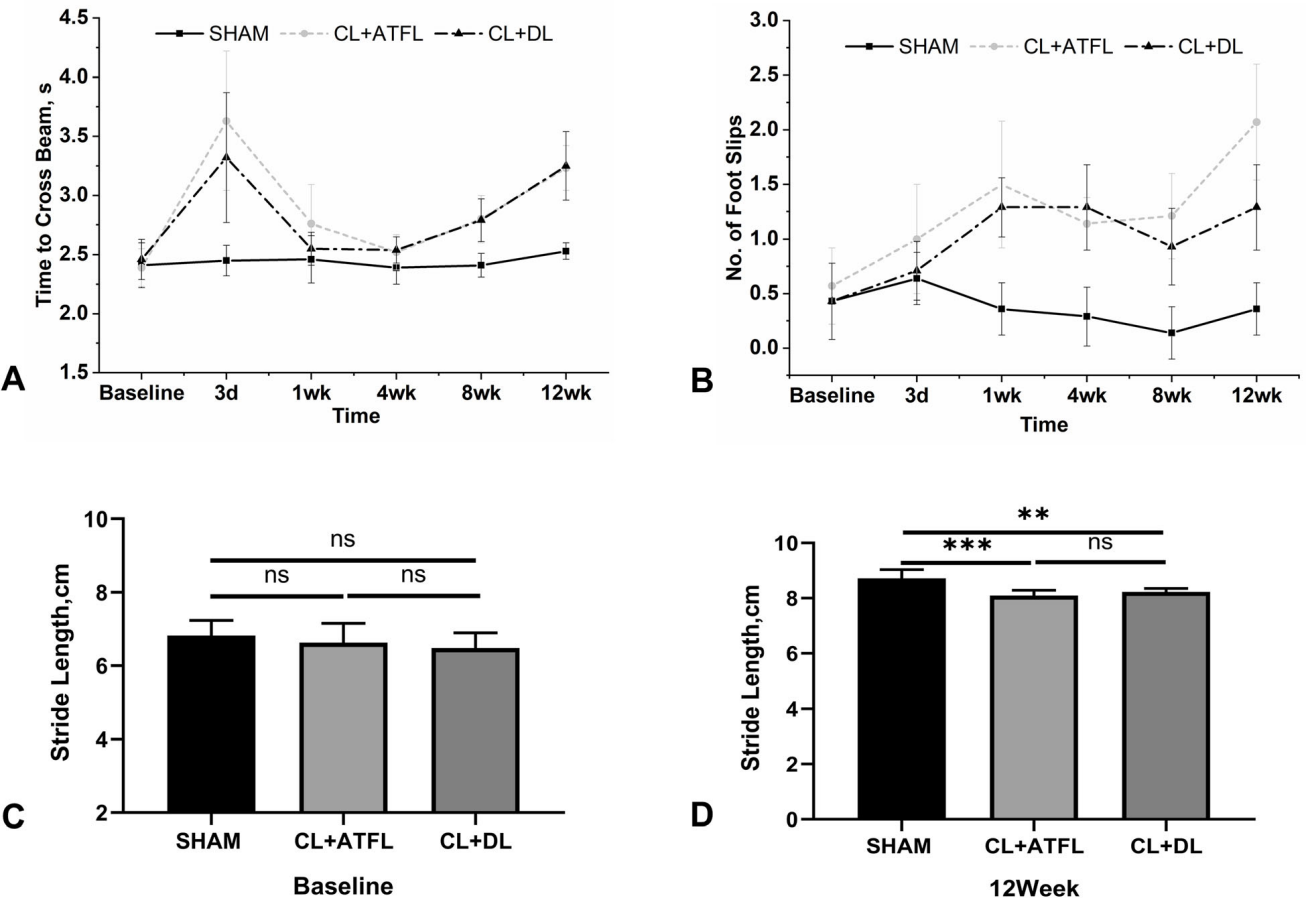
Behavioral analysis of mice, means ± standard deviations. **A** Time required for mice to cross the balance beam. **B** The number of slips of the right foot when traversing the balance beam. **C** Comparison of right footstep length of mice in each group before surgery. **D** Comparison of right footstep length of mice in each group 12 weeks after surgery. Statistically significant differences are indicated by ** where *P* < 0.01 or *** where *P* < 0.001 between the indicated groups

For the balance beam test, there was no significant difference between each group over 6 tests prior to surgery (*P* = 0.73). One week after surgery, there was a significant difference between the CL+ATFL and SHAM groups (*P* = 0.03), but no significant difference between the CL+DL and SHAM groups (*P* = 0.52). After 4 weeks, there was no significant difference between the CL+ATFL group and the SHAM group (*P* = 0.09, 0.06 > 0.05). In other tests, the time required for mice in the ligament transection group to pass the balance beam was longer than that for mice in the SHAM group (*P* < 0.05) (Fig. 2A). In addition, transection of the ligament increased the number of times the mice slipped (Fig. 2B). There was no significant difference in the number of slips in each group prior to surgery (*P* = 0.68). Three days after surgery, there was no statistical difference between the CL+ATFL group and the SHAM group (*P* = 0.07), or the CL+DL group and the SHAM group (*P* = 0.71). At other time points, the number of slips in the ligament transection group was more than in the SHAM group (*P* < 0.05), while the CL+ATFL group was more prone to slipping than the CL+DL group after 12 weeks (*P* < 0.05).

### Gait

Although the step size of mice increased over time, ligament transection modified the step size (Fig. 2C, D). Preoperatively, there was no significant difference in step length of the right hind foot in each group (*P* = 0.4). Twelve weeks after surgery, the right footstep length of the mice in the ligament transection groups was shorter than in the SHAM group (*P* < 0.01), but there was no significant difference between the CL+ATFL group and the CL+DL group (*P* = 0.86).

### Micro-CT

**Fig. 3:**
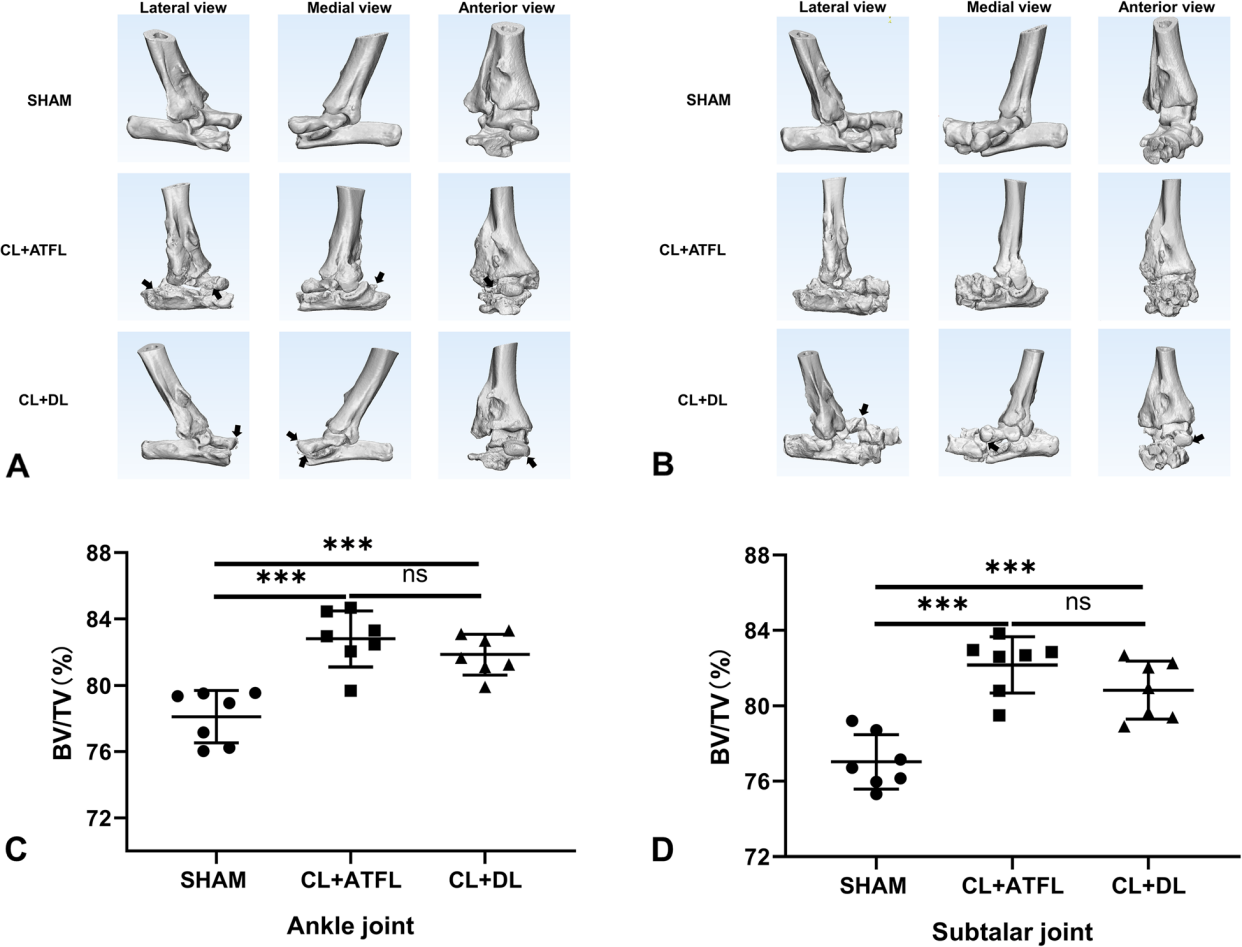
Micro-CT analysis of mouse right feet. **A** Three-dimensional reconstruction of the talus without dislocation in the ankle-subtalar joint complex of mice (lateral view, medial view, anterior view). **B** Three-dimensional reconstruction of dislocated talus in the ankle-subtalar joint complex of mice (lateral view, medial view, anterior view). **C** Quantitative analysis of bone volume fraction (BV/TV) of mouse ankle joints. **D** Quantitative analysis of bone volume fraction (BV/TV) of the subtalar joint in mice. Black arrows indicate osteophyte formation or talus dislocation. Statistically significant differences are indicated by *** where *P* < 0.001 between the indicated groups

The cartilage layer of the ankle-subtalar joint complex was evaluated quantitatively using micro-CT 12 weeks after surgery. As displayed in Fig. 3, during CT image reconstruction, it was found that the ankle-subtalar joint complex of the surgical groups was coarser than that of the SHAM group, with a greater extent of wear and osteophyte formation, suggesting that the instability in the ankle-subtalar joint complex in mice increases wear in the joint and causes degeneration. Approximately 28.6% of the mice in the CL+DL group developed talus dislocation. Furthermore, the bone volume fraction (BV/TV) of the CL+ATFL and CL+DL groups was significantly higher than that of the SHAM group (*P* < 0.01), indicating that cartilage degeneration had occurred in the surgical groups after 12 weeks.

### Analysis of tissue staining

**Fig. 4:**
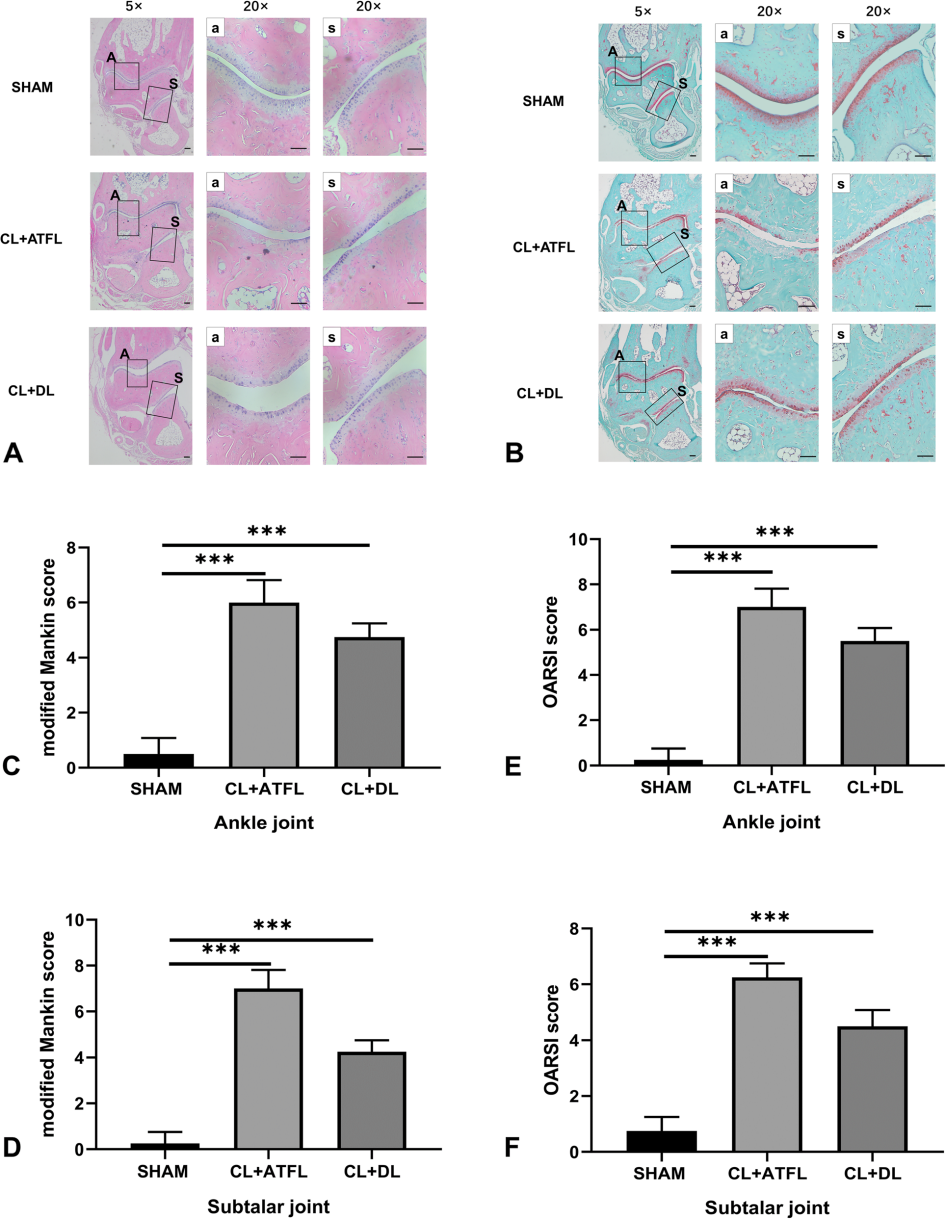
HE and Safranin O-Fast green staining and analysis of the ankle joints. **A** HE staining of the ankle-subtalar joint in mice. **B** Safranin O-Fast staining of the ankle-subtalar joints in mice. **C** Modified Mankin scores for the ankle joints in mice. **D** Modified Mankin scores for the subtalar joints in mice. **E** Osteoarthritis Research Society International (OARSI) scores for the ankle joints in mice. **F** OARSI scores for the subtalar joints in mice. a, ankle joint; s, subtalar joint. Statistically significant differences are indicated by *** where *P* < 0.001 between the indicated groups. Scale bar = 100 μm

To further confirm that ankle-subtalar joint complex instability can cause joint degeneration, HE and Safranin O-Fast Green staining were used to identify cartilage degeneration in the mice. As displayed in Fig. 4, the structure of the ankle-subtalar joint complex in the SHAM group was complete, with undamaged cartilage morphology, with no apparent loss of articular cartilage proteoglycans. Proteoglycan loss from the ankle-subtalar joint complex in the CL+ATFL and CL+DL groups and a rough cartilage surface layer were clearly apparent. OARSI and modified Mankin scores, used to evaluate the three groups, were significantly higher in the ligament transection groups than in the SHAM group (*P* < 0.01).

## Discussion

An ankle sprain is one of the most common injuries presenting in the emergency room, usually accompanied by ligament relaxation or rupture [27]. This type of ligament injury may unbalance the forces in the ankle ligament system, resulting in uneven force distribution around the ankle-subtalar joint complex, resulting in ankle-subtalar joint complex instability [28]. However, researchers remain uncertain about the factors causing ankle-subtalar joint complex instability. Therefore, in the present study, the CL and ATFL or CL and DL were transected in mice, thereby constructing two animal models of ankle-subtalar joint complex instability. In addition to beam balance and gait tests, micro-CT analysis and tissue section staining were also used to verify whether long-term ankle-subtalar joint complex instability developed into PTOA. The results indicated that the balance and gait of mice were impaired by transection of these ligaments, and therefore is an effective animal model of ankle-subtalar joint complex instability, which if not treated in time gradually causes articular cartilage degeneration.

### Ligament protocol

In the ligaments surrounding the ankle-subtalar joint complex, isolated CL injuries are rare, as they are usually accompanied by joint injuries of the ankle ligaments [29]. The ATFL is the most vulnerable ligament in the lateral ankle ligament, but the role of the ATFL in stabilizing the ankle-subtalar joint complex is controversial because early biomechanical studies have indicated that transection of the ATFL alone has no apparent effect on the movement of the ankle-subtalar joint complex, although a number of researchers have pointed out that ankle-subtalar joint complex instability can be caused by injury to the lateral and subtalar ligaments [1, 4, 30]. In addition, a number of studies have shown that approximately 40% of patients treated for ankle instability display DL injury. Although some researchers continue to dispute whether such DL injuries should be repaired, some believe that the quality of life of patients is unaffected in the absence of a repair [31, 32]. Therefore, we aimed in the present study to explore whether joint injury from transection of the CL+ATFL or CL+DL ligaments affected ankle-subtalar joint complex instability, ultimately reducing the quality of life of a patient, and to provide an updated reference for clinical treatments of ankle-subtalar joint complex instability.

### Balance beam and gait

Three days following surgery, the time required to cross the balance beam by the CL+ATFL group was 51.9% longer than that prior to surgery and 35% longer for the CL+DL group. No difference was observed for the SHAM group. The increased time may be due to the decreased level of exercise in the mice after acute injury, similar to the decreased exercising capability in humans after acute injury. Eight weeks after surgery, the time required to cross the balance beam in the CL+ATFL group was 17.2% longer and 13.4% longer in the CL+DL group than prior to surgery, and again unchanged for the SHAM group. The increased time required by the ligament transection groups at this time point suggests that the cartilage may have started to degenerate, the pain slowing down the mice crossing the balance beam. Twelve weeks after surgery, the time required for the CL+ATFL group increased by 35.1% for the beam balance assay and 32.1% in the CL+DL group, while the time increased by 5% in the SHAM group. At this time point, the motion of the ankle in the ligament transection group was smaller than that 8 weeks after surgery, and it was more rigid, with joint degeneration becoming more apparent. The increased times at 8 and 12 weeks are consistent with observations by Hubbard-Turner after transection of the lateral ankle ligament [15, 19]. However, the results were different 3 days after surgery [15]. The reason for the difference may be that the lateral ligament of the ankle joint is relatively shallow, and the trauma caused by transection is somewhat less. After 3 days, the ankle was not inflamed and was without apparent swelling, and so crossing the barrier was not impaired. The CL is located deep within the joint, and at least two ligaments are transected at the same time in the ligament transection groups. This induces a large degree of trauma, with swelling that has not been relieved within 3 days. Walking causes pain, and so activities requiring movement take longer. However, there was almost no swelling after surgery in the SHAM group, and the time to perform the activity did not increase.

In the slip test, the mice in the ligament transection group were more likely to slip than in the SHAM group. After 12 weeks, the number of occasions the mice slipped in the ligament transection groups was greater than that prior to surgery. The number of times in the CL+ATFL group increased by 3.6-fold, and 3-fold in the CL+DL group, while it decreased by 16.3% in the SHAM group. In addition, the number of slips in the CL+ATFL group 12 weeks after surgery was 1.6-fold that of the CL+DL group. The results suggest that instability in the ankle-subtalar joint complex occurred in the two ligament transection groups, with loss of the lateral ligament more likely to cause slipping than that of the medial ligament. The number of slips in the ligament transection group was similar to that observed by Hubbard-Turner, but the number of slips in the SHAM group did not differ from that in the Hubbard-Turner group [15, 19]. The reason for the difference may be that the mice were trained before the test to ensure they were familiar with the balancing task. As there was no instability in the SHAM group, no increased slipping occurred.

In the gait test, the length of the steps in each group increased over time. Twelve weeks after surgery, the step size of the CL+ATFL group was 7.2% smaller than that of the SHAM group and 5.6% smaller in the CL+DL group compared with the SHAM group. This suggests that instability of the ankle-subtalar joint complex caused by ligament transection can impair normal gait in mice. The results are similar to those of Hubbard-Turner who found that transection of the lateral ankle ligament impaired the gait of the mice [19].

### Micro-CT

Micro-CT analysis 12 weeks after surgery demonstrated that the volume fraction of the ankle bone in the CL+ATFL group was 6% greater than that of the SHAM group and 4.8% greater in the CL+DL group than in the SHAM group. The ankle bone volume fraction in the subtalar joint in the CL+ATFL group was 6.7% higher and 4.9% higher in the CL+DL group than in the SHAM group. These results suggest that the cartilage in the ankle-subtalar joint had degenerated in the ligament transection groups. CT image reconstruction demonstrated that the surfaces in the ankle-subtalar joint complex in the ligament transection groups were rougher than in the SHAM group, with osteophytes clearly present. This is similar to CT analysis performed by Chang et al., who established an ankle OA model by surgery [16].

### HE and Safranin fast green

HE staining indicated that the number of chondrocytes on the surface of the ankle-subtalar joint complex in the two groups declined or they were absent, and the structure had started to degenerate. This was also verified by Safranin O-Fast Green staining, which suggested that failing to treat ankle-subtalar joint complex instability in time resulted in degeneration of the articular cartilage, similar to the conclusions of Chang et al. [16].

### Adjacent joint degeneration

Interestingly, in 3D reconstructed CT images in the present study, it was found that the talonavicular joint surface in the ligament transection group was coarser than that in the SHAM group, suggesting that it may have degenerated. This is consistent with the previous findings that severe ankle instability can cause adjacent joint degeneration, which indicates that the extent of instability in this animal model was considerable, altering the biomechanics around the ankle and affecting the uniform stress of adjacent joints [9]. In addition, ankle arthrodesis, an effective treatment for severe osteoarthropathy, still changes the musculoskeletal biomechanics, thus increasing the risk of adjacent joint degeneration [33]. Although Ilizarov fixation is superior to internal fixation, restoration of the biomechanical balance of the musculoskeletal system around the foot and ankle will be the focus of treatment in the future [10].

### Limitations

The study has a number of limitations. OA is a chronic progressive disease. An experiment of a longer duration is required to observe the relationship between joint instability and OA. Additionally, the layer of cartilage in the ankle-subtalar joint is too thin in mice to obtain sufficient cells for in vitro experimentation to evaluate the changes in inflammatory factors. The study was performed on a small group of animals, and there was a relatively short period from the beginning of the study to histological studies. In the future, it is worth conducting research on a larger number of animals over a longer duration. In addition, the mechanism of joint degeneration using molecular biology is worth studying.

## Conclusions

Transection of the CL+ATFL or CL+DL causes mechanical instability of the ankle-subtalar joint complex in mice, leading to decreased balance and shortening of the length of the step. In addition, this mechanical instability would also lead to dislocation of the talus, accelerate wear, and loss of the articular cartilage, resulting in the development of PTOA. The present study successfully established an animal model to simulate the changes in human behavior after acute ankle-subtalar ligament injury in mice, which represents a quantitative approach to the early diagnosis of ankle-subtalar joint complex instability. It will also allow researchers to better understand how an acute musculoskeletal injury gradually develops into articular cartilage degeneration.

## Data Availability

The datasets used and/or analyzed during the current study are available from the corresponding author on reasonable request.

## Abbreviations

PTOA: Post-traumatic osteoarthritis
SHAM group: Sham surgery group
CL+ATFL group: Transected cervical ligament + anterior talofibular ligament group
CL+DL group: Transected cervical ligament + deltoid ligament group
CL: Cervical ligament
ATFL: Anterior talofibular ligament
DL: Deltoid ligament
OA: Osteoarthritis
ROI: Region of interest
HE: Hematoxylin and eosin
SD: Standard deviation
BV/TV: Bone volume fraction
